# Results of health technology assessments of orphan drugs in Germany—lack of added benefit, evidence gaps, and persisting unmet medical needs

**DOI:** 10.1017/S026646232400062X

**Published:** 2024-12-03

**Authors:** Philip Kranz, Natalie McGauran, Can Ünal, Thomas Kaiser

**Affiliations:** 1 Researcher, Drug Assessment Department, Institute for Quality and Efficiency in Health Care (IQWiG), Cologne, Germany; 2 Researcher, Communications Department, IQWiG, Cologne, Germany; 3 Director, IQWiG, Cologne, Germany

**Keywords:** orphan drugs, health technology assessment, added benefit

## Abstract

**Background:**

The number of orphan drug (OD) approvals has increased sharply in Europe. In Germany, all ODs are initially subject to a limited assessment after market access. Their added benefit over the standard of care is accepted as established upon EU approval; a regular health technology assessment (HTA) is performed only in certain cases.

**Objective:**

We assessed whether the increase in OD approvals has led to improvements in patient-relevant outcomes as supported by the evidence submitted by market authorization holders (MAHs) for HTA in Germany. We also examined the extent to which these ODs address unmet medical needs.

**Methods:**

The results of limited assessments and regular HTAs of ODs in Germany (January 2011–September 2021, plus January–December 2023) were analyzed to determine their added benefit based on MAH-submitted dossiers. Added benefit was reported separately for each research question generated from the EU-approved therapeutic indications and any sub-indications (e.g., different subpopulations or control interventions) specified for HTA in Germany.

**Results:**

Eighty-nine ODs (limited assessments: sixty-nine; regular HTAs: twenty) were evaluated in 175 research questions (limited assessments: ninety-seven; regular HTAs: seventy-eight). The added benefit granted in limited assessments was non-quantifiable in nearly eighty percent of the ninety-seven questions. In regular HTAs, no proof of added benefit was shown in fifty-four percent of the seventy-eight questions, mainly due to insufficient comparative data with the standard of care. Established treatments were available for fifty-eight percent of the seventy-eight questions; more than half of which addressed oncology indications (although these account for only eight percent of rare diseases).

**Conclusions:**

Due to evidence gaps in post-approval HTA, many ODs approved in the EU lack proof of added benefit in terms of improving patient-relevant outcomes. Moreover, most approved ODs are indicated for diseases with established treatments and oncology indications, while many unmet medical needs remain. Incentives are required to encourage research in areas of unmet medical need and to generate comparative data with the standard of care.

## Introduction

### Orphan drugs in the European Union

In the European Union (EU), rare diseases are those with a prevalence of less than or equal to five per 10,000 population (1). There are often no or inadequate treatments for these diseases. To create incentives for the pharmaceutical industry to invest in the development of drugs for rare diseases (orphan drugs (OD)s) despite market risks, the Regulation on Orphan Medicinal Products (in short: Orphan Regulation) was introduced in 2000 (2). A key incentive of this regulation is market exclusivity, which guarantees that, with few exceptions, no similar ODs will be approved for the same therapeutic indication (i.e., the indication defined in the EU Summary of Product Characteristics) for the next ten years (or twelve years in the case of pediatric diseases).

Since the introduction of the Orphan Regulation, there has been a steady increase in the number of OD approvals: sixty-three ODs were approved in the EU in the first decade after 2000, 133 in the second, and thirty-five in 2022/2023 (3). To improve patient access to ODs at the national level, most European countries have introduced further measures (e.g., special patient access schemes, and exclusion from regular assessment) (4).

### Orphan drugs in early benefit assessments in Germany

Since the introduction of the German Act on the Reform of the Market for Medicinal Products (AMNOG) in 2011, most newly approved drugs (and established drugs approved for new therapeutic indications) must undergo an “early benefit assessment” after market access. These regular health technology assessments (HTAs) determine the added benefit of a drug in terms of an improvement in patient-relevant outcomes compared with the current standard of care in Germany. Six rating categories are possible for a regular HTA: If an added benefit is shown, its extent is classified as “minor,” “considerable,” “major,” or “non-quantifiable”; if there is no added benefit, the drug is classified as “added benefit not proven” or “less benefit.”

However, ODs are not subject to this procedure. Their added benefit is accepted as established upon EU approval. The Federal Joint Committee (G-BA), the main decision-making body in the German health care system, assesses the evidence submitted by the MAH. However, no standard of care is specified in these limited assessments and only four rating categories are possible. If the added benefit cannot be classified as “minor,” “considerable,” or “major,” the G-BA automatically classifies the drug as having a “non-quantifiable” added benefit. The categories “added benefit not proven” or “less benefit” are omitted.

**Figure 1.**
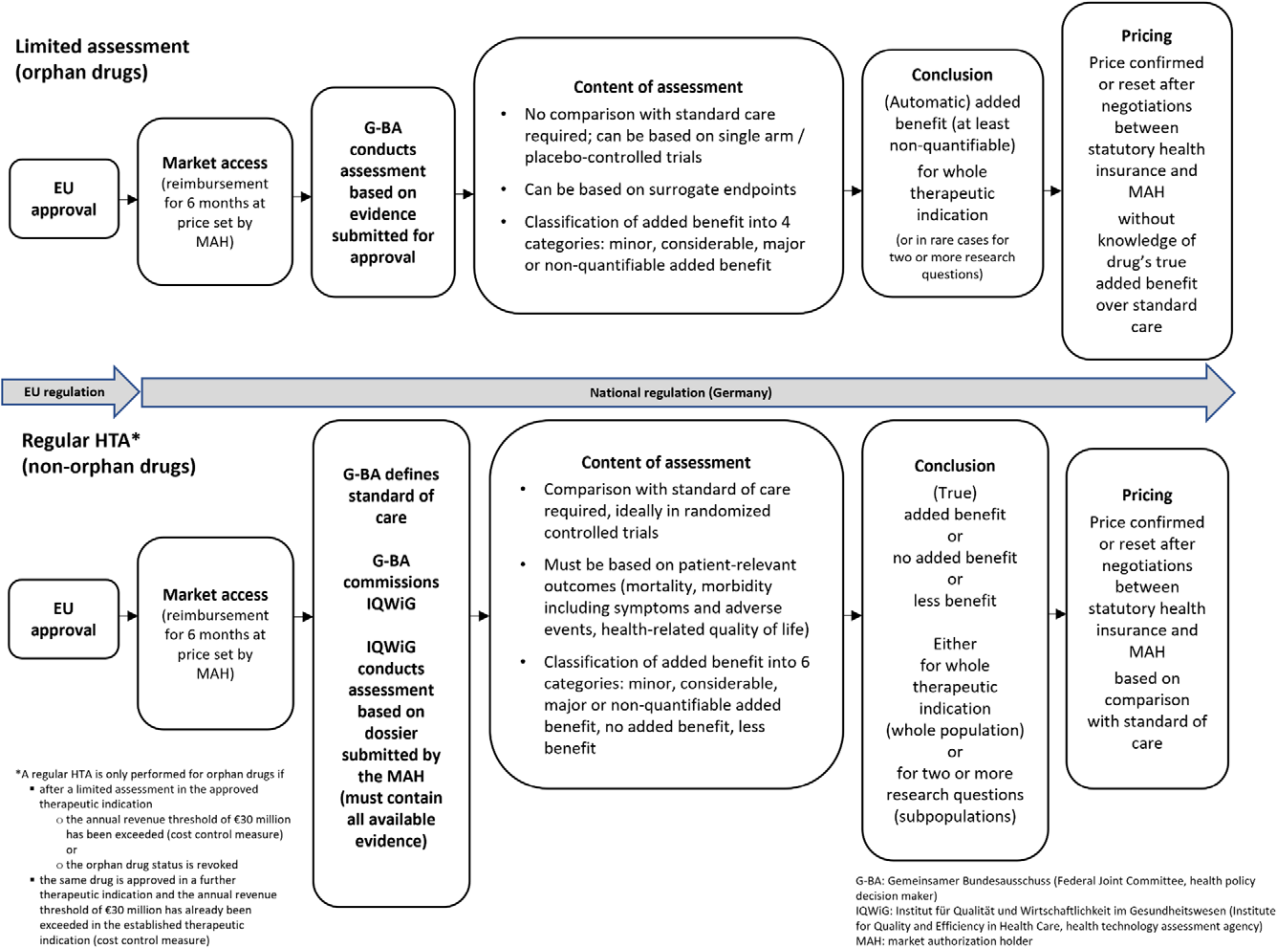
Limited assessments and regular HTAs in Germany.

For most newly approved drugs, the results of regular HTAs and limited assessments inform price negotiations between the MAH and the umbrella organization of the statutory health insurance funds. This price applies from six months after market access; the MAH sets the price for the first six months (see Figure 1 comparing limited assessments and regular HTAs).

A regular HTA of an OD is only conducted if the annual sales threshold, currently thirty million euros (fifty million euros before January 2023), is exceeded in the previous twelve months or if the OD status is revoked. In regular HTAs, and less frequently in limited assessments, the G-BA may divide the approved therapeutic indication of a drug into two or more sub-indications (e.g., different subpopulations or control interventions) to account for specific populations treated with a different standard of care in Germany. The questions generated from the EU-approved therapeutic indications and any sub-indications specified by the G-BA are called “research questions.”

### Recent developments

The European Commission’s evaluation of the Orphan Regulation in 2020 found that, while the legislation has supported the development and availability of ODs as intended, this often applies to more profitable areas; it has only been partly successful in stimulating drug development in areas of high unmet medical need (1).

The Institute for Quality and Efficiency in Health Care (IQWiG), the German HTA agency, recently posted a working paper (5) on its website analyzing the added benefit of ODs that had undergone regular HTA as well as a related health policy journal article (4). In addition to presenting the main findings of the working paper, this article presents additional analyses: the added benefit of ODs determined in limited assessments, the type of control intervention used in the studies for regulatory approval, and the type of therapeutic indication for which the drug was approved.

### Rationale and aim of the study

Despite a sharp increase in the number of OD approvals, it is unclear to what extent patients have actually benefited from this development. This study assessed the extent to which ODs approved in the EU in the last decade led to improvements in patient-relevant outcomes as supported by the evidence contained in MAH dossiers submitted for HTA in Germany. We also examined the extent to which these ODs addressed unmet medical needs.

## Methods

### Information retrieval

We analyzed all ODs that underwent at least one limited assessment after market access between 1 January 2011 and 30 September 2021. All subsequent regular HTAs of these drugs conducted up to 30 September 2021 were also included, provided that the EU OD status still applied. To ensure completeness, the drugs were identified via the G-BA’s HTA database using the OD filter (6).

### Information synthesis

The OD assessments were divided into limited assessments and regular HTAs. The results of the assessments were categorized in terms of added benefit compared with the available standard of care according to the decisions of the G-BA. There is always a standard of care, even if it is only best supportive care (BSC) or watchful waiting. Additional analyses were conducted to assess the impact of different factors on the likelihood of added benefit: the type of therapeutic indication (oncology vs. non-oncology) and the type of control intervention defined by the G-BA (active control vs. BSC or watchful waiting). The latter step also identified areas of unmet medical need. Finally, the evidence base submitted in the MAH dossiers and used in the regular HTAs was divided into four categories of study design (in descending order of level of evidence): randomized controlled trials (RCTs), adjusted indirect comparisons, non-RCTs, and no (usable) data. If more than one study design was presented in the MAH dossier, the study design with the highest level of evidence was considered. The results on added benefits were presented separately for each analysis.

Our analysis was based on the data pool contained in the above-mentioned working paper (5), which includes assessments conducted over almost eleven years, between January 2011 and 30 September 2021. To assess the impact of the reduction in the revenue threshold for regular HTAs of ODs (effective from 1 January 2023), in June 2024 we conducted an analysis of regular HTAs of ODs completed in 2023 to check, through a sensitivity analysis, whether their inclusion affected the results of the original analysis.

## Results

### Information retrieval

Eighty-nine ODs and their associated 175 eligible research questions were identified that had undergone at least one limited assessment between January 2011 and September 2021 (Supplementary Figure S1). Sixty-nine of these drugs (seventy-eight percent) and their associated ninety-seven research questions only underwent a limited assessment; twenty of the eighty-nine drugs (twenty-two percent) and their associated seventy-eight questions later also underwent a regular HTA.

### Limited assessments

**Figure 2.**
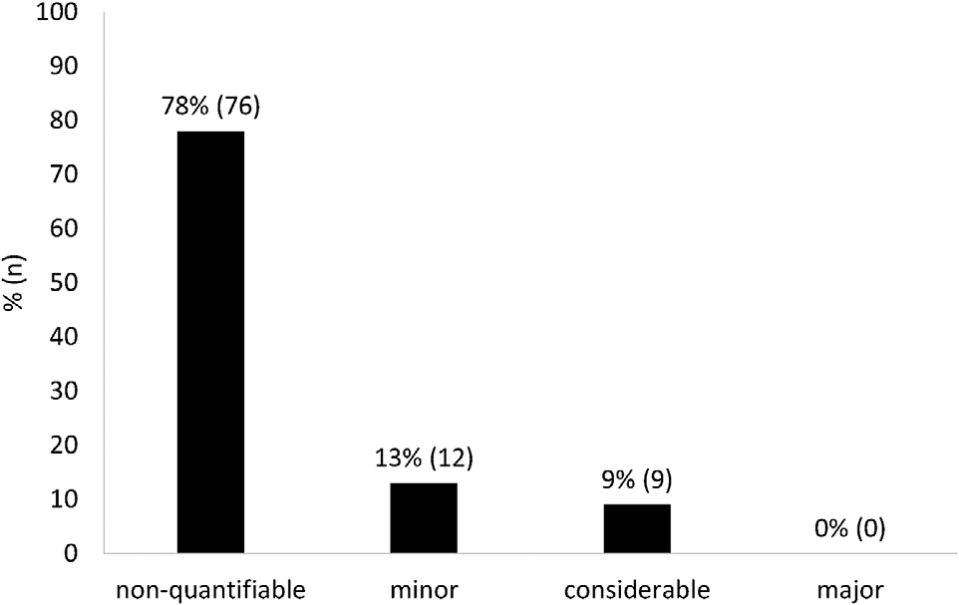
Extent of added benefit in limited assessments of orphan drugs.

For seventy-eight percent (*n* = 76) of the ninety-seven research questions analyzed in limited assessments, the added benefit could not be quantified. For the remaining twenty-one questions, the added benefit was classified as either minor or considerable (Figure 2).

### Regular HTAs

#### Added benefit, overall

**Figure 3.**
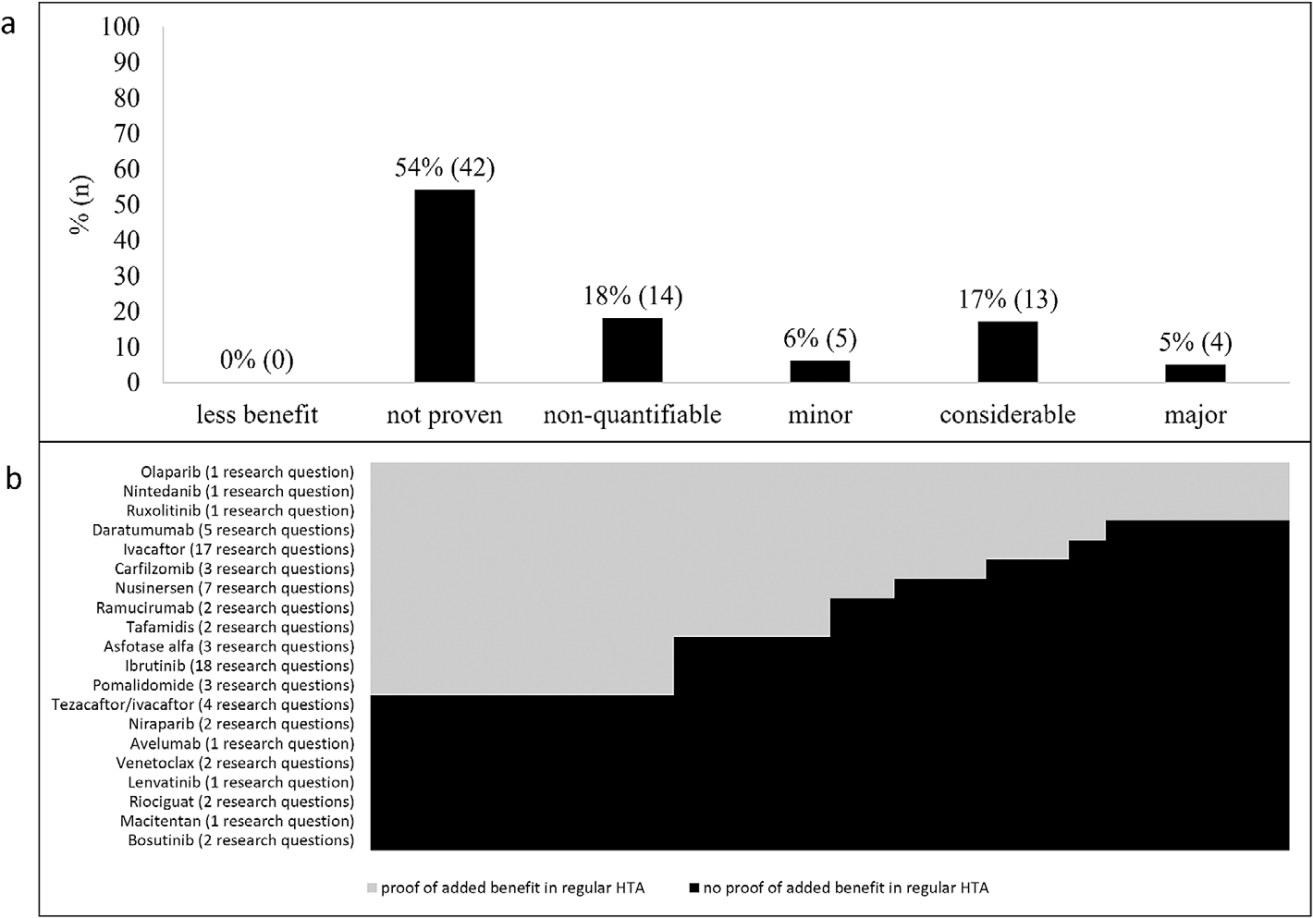
Distribution of ratings of added benefit from regular HTAs of orphan drugs: (a) Summary (b) Individual drugs. Each bar equals 100 percent of the research questions examined. Both graphs are reproduced from the IQWiG working paper (5).

For fifty-five percent (*n* = 42) of the seventy-eight research questions analyzed in regular HTAs, no added benefit was shown (Figure 3a). At the drug level, only fifteen percent (*n* = 3) of the twenty ODs (ruxolitinib, nintedanib, and olaparib) showed an added benefit in all the questions analyzed. The remaining eighty-five percent (*n* = 17), showed no added benefit in at least one question (Figure 3b).

#### Control interventions

**Figure 4.**
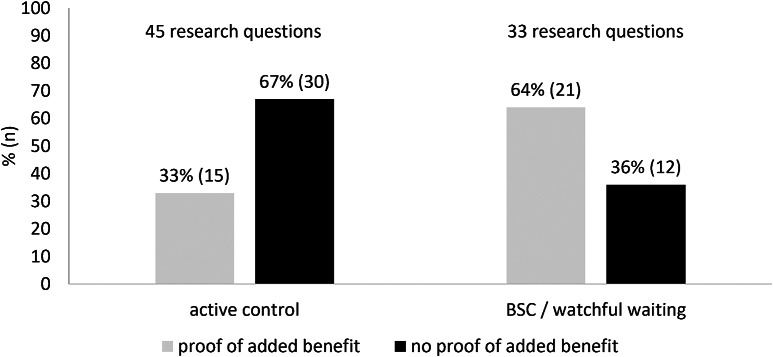
Results on added benefit from regular HTAs of orphan drugs according to type of control intervention.

Active controls were available for fifty-eight percent (*n* = 45) of the seventy-eight research questions; for the remaining forty-two percent (*n* = 33), only BSC or watchful waiting was available (Figure 4). In comparisons of ODs with BSC or watchful waiting, proof of added benefit was almost twice as likely for comparisons of ODs with BSCs or watchful waiting versus comparisons of ODs with active controls.

#### Therapeutic indications

**Figure 5.**
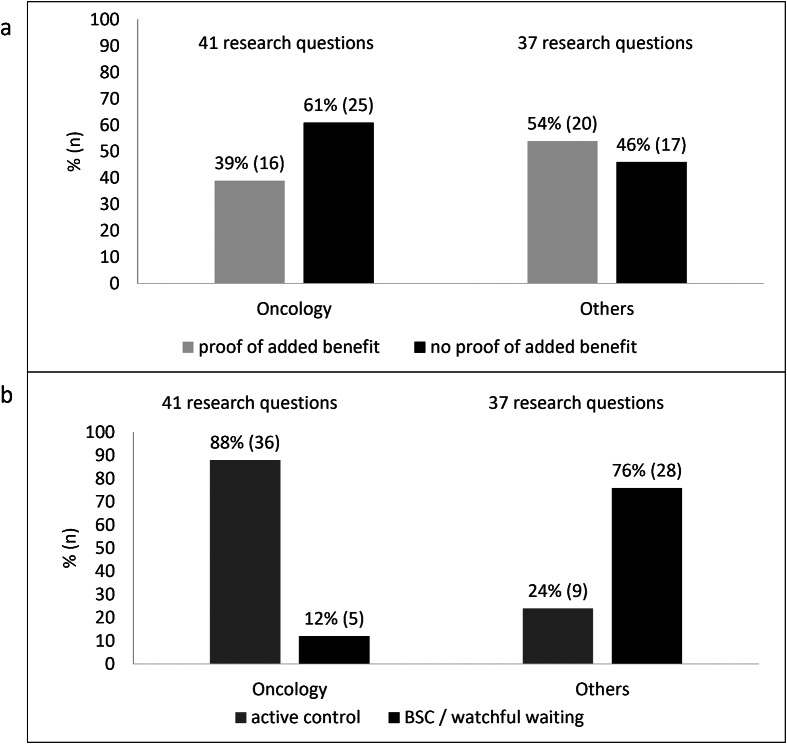
Regular HTAs for orphan drugs: oncology versus other indications: (a) Results on added benefit (b) Type of control intervention.

Fifty-three percent (*n* = 41) of the research questions for regular HTAs in oncology indications (Figure 5a). Proof of added benefit was less likely in these indications than in other diseases (thirty-nine percent vs. fifty-four percent of questions).

Whereas eighty-eight percent (*n* = 36) of the questions in oncology had an available active control at the time of the regular HTA, only twenty-four percent (*n* = 9) of the questions in other indications had an active control as the standard of care defined by the G-BA (Figure 5b).

#### Type of evidence

In almost half of the cases of regular HTAs, no (usable) data were available. This was also the most common reason for the conclusion “added benefit not proven” (Supplementary Table S1). Data from RCTs were available for thirty-six percent (*n* = 28) of the seventy-eight research questions. The quantification of the added benefit into “minor,” “considerable,” or “major” was based almost exclusively on RCTs, with the exception of one question.

#### Sensitivity analysis of orphan drugs assessed in regular HTAs completed in 2023

In 2023, twenty-six research questions on ODs were investigated in regular HTAs. For all of these questions, a decision by the G-BA on the added benefit was available in June 2024: for one (four percent) question the added benefit was less, for seventeen (sixty-five percent) questions, the added benefit was not proven, for six (twenty-three percent) it was non-quantifiable, for one (four percent) it was minor and for one (four percent) it was considerable.

## Discussion

Many ODs assessed in regular HTAs lack proof of added benefit. In this respect, ODs fare no better than non-ODs (7). The added benefit accepted as established upon EU approval for the limited assessments was found to be non-quantifiable in almost eighty percent of cases. Unlike regular HTA, there are no defined standards for the evidence submitted for ODs subject to limited assessment; it is unclear what proportion of these limited assessments had no data comparing the ODs to the standard of care. However, based on the results of the regular HTAs, it is likely that most of the cases of “non-quantifiable” added benefit were cases where there was no proof of added benefit due to a lack of such data, although other reasons (e.g., study duration too short, lack of patient-relevant endpoints) may also apply. The results of the sensitivity analysis (including all regular HTAs completed in 2023) were consistent with the results of the original analysis and showed that the observed pattern for regular HTAs of ODs remained unchanged: for most research questions, no added benefit was demonstrated.

Our analysis showed that patients with rare diseases without established treatments and with non-oncology indications particularly benefit from new ODs, as the likelihood of added benefit (i.e., relative to BSC or watchful waiting) is higher than in diseases with established treatments and oncology indications. However, established treatments were already available for more than half of the research questions included, indicating a lack of OD approvals in areas of unmet need. In addition, ODs were clustered in oncology indications (which typically generate higher revenues (8)), even though oncology indications account for less than ten percent of all rare diseases (9). These findings are consistent with those of the European Commission that “clustering of products is observable in some areas, while in others, research and development is wholly absent, leaving high unmet needs” (1).

Although RCTs are available for about two-thirds of approved OD indications (5), they often lack comparisons with the standard of care, and are therefore of limited use in HTA. While this finding suggests that RCTs in rare diseases are largely feasible, it also highlights a suboptimal use of resources. As RCTs in rare diseases are logistically challenging due to small sample sizes (10), the evidence generated by RCTs for regulatory approval should be maximized to answer questions relevant to clinical and health policy decision-making. This is particularly important, as the evidence gaps at market access are usually not filled later, and new RCTs to generate comparative data with the standard of care are rarely conducted after market access (5). This calls for reform of evidence requirements for OD approval. To inform clinical and health policy decisions, comparative data with the best available standard of care should be generated and submitted for regulatory approval or required shortly after regulatory approval as, for example, a condition of continued market access. Incentives to generate such evidence on a timely basis should be considered, as appropriate (11). Furthermore, the current research infrastructure for rare diseases should be improved with, for example, innovative trial designs, selection of endpoints, statistical analysis, and greater use of patient perspectives (12;13). Sample sizes can be increased by establishing international rare disease registries, which could play an important role in generating comparative evidence if the current infrastructure is strengthened and data quality is improved (14). It should be noted that since 2020, the G-BA has been able to require MAHs to generate post-marketing evidence, but this requirement is limited to non-randomized trials and results will be available only after several years.

The unit or per-patient costs of ODs, which are much higher than those of non-ODs, should also be taken into account (15;16). Overcompensation is a potential contributing factor, which has been identified by the European Commission as another negative effect of the Orphan Regulation (1). Although spending on ODs is still relatively low in relation to total drug spending, it is significantly disproportionately high (17;18). An analysis of the German drug market in 2020 showed that while ODs represented only 0.06 percent of all prescriptions, they accounted for 11.6 percent of total drug spending (17). OD development is partly subsidized by public funding and the costs of prescribed ODs are largely reimbursed by taxpayer- or member-financed health insurance funds. Therefore, on behalf of patients with rare diseases and society as a whole, it is both an ethical and financial responsibility to ensure that ODs provide an added benefit over the standard of care.

Although many of the above issues have been addressed by the European Commission’s proposals to incentivize OD development (19), there are several key uncertainties that diminish the likelihood of these proposals resolving the challenges described here. IQWiG has made several proposals for the revision of the EU pharmaceutical legislation, such as measures to incentivize the conduct of comparative studies and to improve data and research infrastructure (20).

## Conclusion

Many ODs that are approved for market access in the EU lack proof of added benefit in post-approval HTA due to evidence gaps, including a lack of comparisons with the available standards of care. This contributes to uncertainty in health policy (e.g., pricing) and clinical decisions. Moreover, OD approvals, and presumably OD development, tend to focus on areas with established treatments, particularly for oncology indications, and less on areas of high unmet medical need. This calls for incentives to pursue research in such areas, including the generation of comparative data with the standard of care for submission to the approval process, to better inform clinical and health policy decision-making.

## Supporting information

Kranz et al. supplementary material Kranz et al. supplementary material

## Supplementary material

The supplementary material for this article can be found at http://doi.org/10.1017/S026646232400062X.
