# Supplementary material for: Results of health technology assessments of orphan drugs in Germany—lack of added benefit, evidence gaps, and persisting unmet medical needs
Source: Int J Technol Assess Health Care. 2024 Dec 3;40(1):e68. doi: 10.1017/S026646232400062X (PMC11703625; doi:10.1017/S026646232400062X)
Supplement: Kranz et al. supplementary material [file S026646232400062Xsup001.docx]

Figure S1


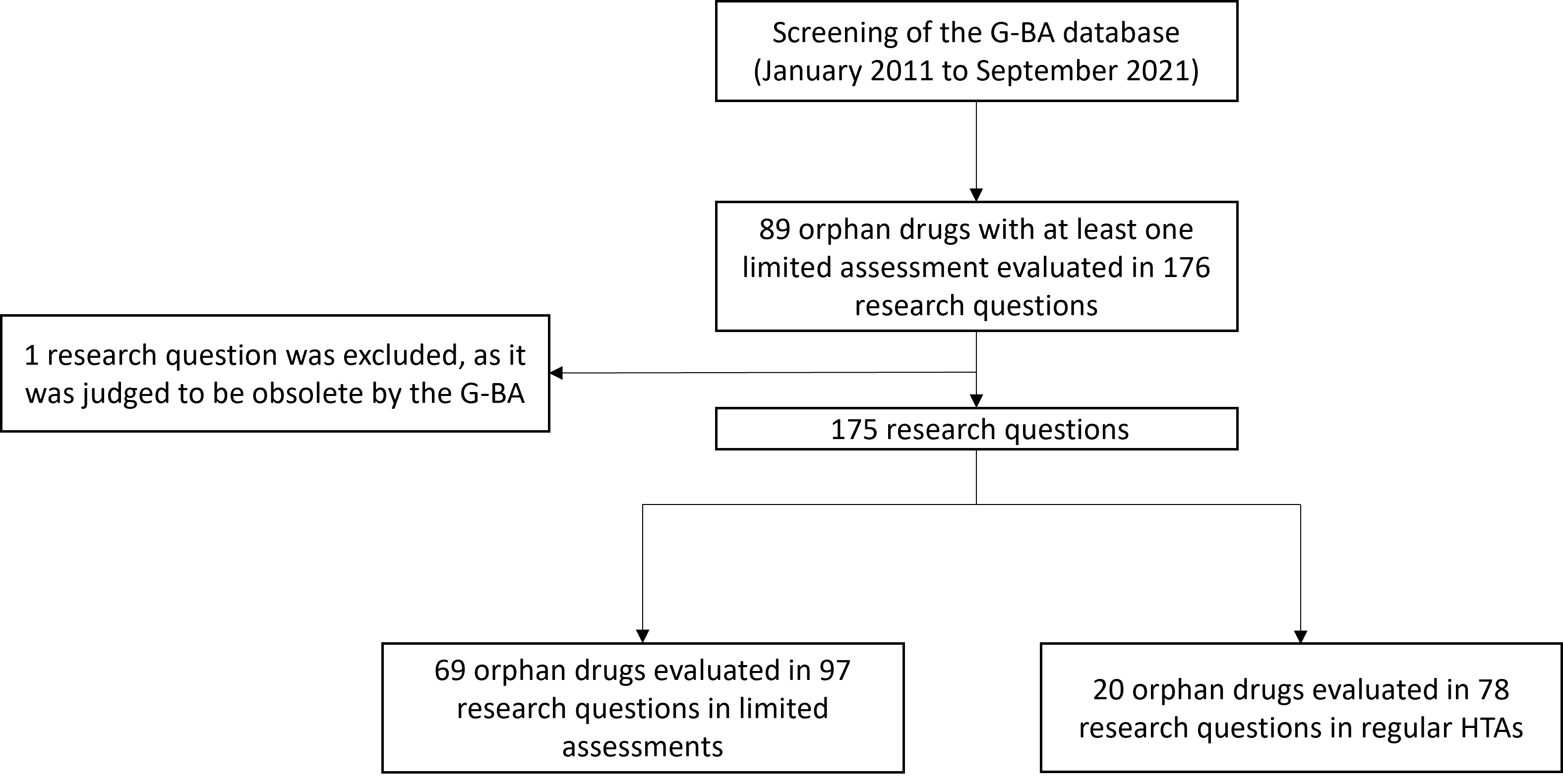


Table S1

|  | | | | | | |  |
| --- | --- | --- | --- | --- | --- | --- | --- |
|  | | **Research questions % (n)** | | | | |  |
| **Data basis**    **Added benefit** | **RCT** | | **Adjusted indirect comparison** | **Non-RCT** | **Evidence transfer** | **No (usable) data** | **Sum** |
| Less benefit | 0% (0) | | 0% (0) | 0% (0) | 0% (0) | 0% (0) | **0% (0)** |
| Not proven | 3% (2) | | 4% (3) | 0% (0) | 0% (0) | 47% (37) | **54% (42)** |
| Non-quantifiable | 6% (5) | | 0% (0) | 1% (1) | 10% (8) | 0% (0) | **18% (14)** |
| Minor | 6% (5) | | 0% (0) | 0% (0) | 0% (0) | 0% (0) | **6% (5)** |
| Considerable | 17% (13) | | 0% (0) | 0% (0) | 0% (0) | 0% (0) | **17% (13)** |
| Major | 4% (3) | | 0% (0) | 0% (0) | 1% (1) | 0% (0) | **5% (4)** |
| **Sum** | **36% (28)** | | **4% (3)** | **1% (1)** | **12% (9)** | **47% (37)** | **100% (78)** |
| n: number of research questions; RCT: randomized controlled trial | | | | | | |  |
